# *Rickettsia felis* in Fleas, Germany

**DOI:** 10.3201/eid1408.071546

**Published:** 2008-08

**Authors:** Jérémie Gilles, Frank Thomas Just, Cornelia Silaghi, Ingrid Pradel, Lygia Maria Friche Passos, Heidi Lengauer, Klaus Hellmann, Kurt Pfister

**Affiliations:** * Ludwig-Maximilians-University, Munich, Germany; † Universidade Federal de Minas Gerais, Belo Horizonte, Brazil; ‡ Klifovet AG, Munich; 1 Current affiliation: University of Kentucky, Lexington, Kentucky, USA.

**Keywords:** Rickettsia felis, fleas, Germany, dispatch

## Abstract

Among 310 fleas collected from dogs and cats in Germany, *Rickettsia felis* was detected in all specimens (34) of *Archaeopsylla erinacei* (hedgehog flea) and in 9% (24/226) of *Ctenocephalides felis felis* (cat flea). *R. helvetica* was detected in 1 *Ceratophyllus gallinae* (hen flea).

*Rickettsia felis*, the causative agent of the flea-borne spotted fever rickettsiosis, is pathogenic for humans (*1*–*4*). Since the first detection of *R*. *felis* from midgut epithelial cells of the cat flea, *Ctenocephalides felis felis*, in 1990 (*5*), interest in the role of this flea species as its main vector has increased. *R*. *felis* has been found in cat fleas on all continents (*6*). Because *R*. *felis* is not lethal for cat fleas and is transmitted transovarially by these fleas (*4*), *C*. *felis* could be a vector and a reservoir of this pathogen. For these reasons, the cat flea was considered the only flea species with a major role in the epidemiology of flea-borne spotted fever rickettsiosis. However, *R*. *felis* has been reported in other flea species (*4*,*6*–*8*), and flea-borne spotted fever rickettsiosis is now considered an emerging human infectious disease. We analyzed the presence of *R*. *felis* in different flea species collected from naturally infested cats and dogs in different locations in Germany.

## The Study

A total of 310 fleas were collected from 49 dogs and 54 cats in 11 widely distributed locations in Germany (Berlin, Munich, Brandenburg, Leipzig, Chemnitz, Rostock/Laage, Bremen, Osnabrück, Münster, Freising, and Schongau) (Figure) in 2007. Specimens collected were recorded and kept at –20°C. Samples were shipped on dry ice to our laboratory, and species identification was performed by using light microscopy and following the determination key of Hopkins and Rothschild (*9*). Because of infestation variations (1–150 fleas per animal), 3 fleas per animal host were chosen randomly for species differentiation.

**Figure:**
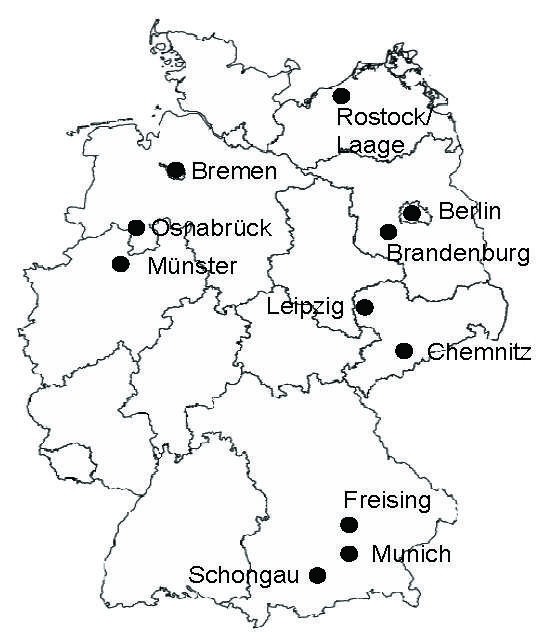
Locations of the 11 flea-collection study sites in Germany, 2007.

Fleas were homogenized individually in 80 μL of phosphate-buffered saline with a RETSCH Tissue Lyser Mixer Mill 300 (QIAGEN, Hilden, Germany) by using 5-mm steel beads. A 100-μL volume of ATL buffer and 20 μL of proteinase K (QIAGEN) were added, and homogenates were incubated at 56°C in an Eppendorf Thermomixer (Eppendorf, Hamburg, Germany) until tissues were completely lysed. DNA was extracted from each flea by using a QIAamp DNA Mini Kit (QIAGEN) according to the manufacturer’s instructions (tissue protocol) and stored at –20°C until used.

PCR amplification of rickettsial DNA was performed by using oligonucleotide primer pairs Rp CS.877p/Rp CS.1258n (*10*) generated from the rickettsial citrate synthase (*gltA*) gene. Positive samples were analyzed for a 530-bp portion of the outer membrane protein A (*ompA*) gene with primer pair Rr 190.70p/Rr 190.602n (*10*) and for a 765-bp portion of the *ompB* gene with primer pair 120–1278/120–3599 (*11*). PCR amplification was accomplished in 50-μL volumes containing 5 μL DNA, 30 μL distilled water, 10 μL 5× *Taq* buffer (Roche, Mannheim, Germany), 3 μL 25 mmol/L MgCl_2_ (Roche), 1 μL 10 mmol/L dNTP (Roche), 0.25 μL each primer (100 μmol/L), and 0.5 μL *Taq* polymerase (5U/mL; Roche). Conditions for the *gltA* and *ompA* PCRs were as described by Bertolotti et al. (*12*). Negative and positive controls were included in all PCRs.

All PCR products were separated by electrophoresis on 1.5% agarose gels at 100 V for 60 min and examined under UV light. Positive samples for both genes were purified by using the QIAquick PCR purification kit (QIAGEN) and sequenced by the MWG Biotech Company (Martinried, Germany). Sequences obtained were compared with those of characterized rickettsia in GenBank by using BLAST analysis (www.ncbi.nlm.nih.gov).

Five species of fleas were identified in the study. The most prevalent species was *C*. *felis* (93% of fleas in cats and 78% in dogs) (Table 1). *Archaeopsylla erinacei* (hedgehog flea), was the second most abundant species, with 26 specimens collected from dogs and 8 from cats. A few specimens of *Ctenocephalides canis* (dog flea), *Pulex irritans* (human flea), and *Ceratophyllus gallinae* (hen flea) were also identified (Table 1). Eight dogs had mixed populations of fleas; 5 had *C*. *felis* and *A*. *erinacei*, 2 had *C*. *felis* and *C*. *gallinae*, and 1 had *C*. *felis* and *C*. *canis*. Mixed populations of fleas were also detected in 3 cats; 2 were infested with *C*. *felis* and *A*. *erinacei*, and 1 with *P*. *irritans* and *C*. *felis*.

**Table 1:** *Rickettsia felis* infection in fleas collected from dogs and cats, Germany, 2007*

Flea species	No. (%) *gltA*-positive fleas	
	Dogs	Cats
*Ctenocephalides felis*	114 (10)	152 (14)
*Archeopsylla erinacei*	26 (26)	8 (8)
*Ctenocephalides canis*	4 (0)	–
*Ceratophyllus gallinae*	2 (1)	3(1)
*Pulex irritans*	–	1 (0)
Total	146 (37)	164 (23)

*gltA, citrate synthase.

Thirty-six (25%) of 146 fleas collected from dogs and 24 (15%) of 164 fleas collected from cats were positive for the *gltA* gene. Positive fleas were found in 6 of 11 sampled locations. Proportions of infected fleas collected from dogs ranged from 25% (Berlin) to 56% (Münster), and proportions of infected fleas collected from cats ranged from 10% (Freising) to 100% (Münster) (Table 2).

**Table 2:** Distribution of *Rickettsia felis* in fleas collected from dogs and cats, Germany, 2007*

Location	Animal	No. animals	No. fleas	Flea species	No. (%) *gltA*+	No. animals with/without infected fleas
Berlin	Dog	8	24	20 *Ctenocephalides felis*,† 4 *Archeopsylla erinacei*†	6 (25)	0/8
	Cat	6	18	17 *C. felis*,† 1 *A.erinacei*†	4 (22)	2/6
Brandenburg	Dog	4	12	12 *C. felis*	0	0/4
	Cat	1	3	*C. felis*	0	0/1
Bremmen	Dog	1	2	2 *C. felis*	0	0/1
	Cat	7	21	*C. felis*	0	0/7
Chemnitz	Dog	10	30	21 *C. felis*, 9 *A.erinacei*†	9 (30)	3/10
	Cat	8	24	*C. felis*	0	0/8
Freising	Dog	–	–	–	–	–
	Cat	7	21	20 *C. felis*,† 1 *A.erinacei*†	1 (5)	2/7
Leipzig	Dog	–	–	–	–	–
	Cat	2	6	*C. felis*	0	0/2
Munich	Dog	7	21	10 *C. felis*, 10 *A.erinacei*,† 1 *Ctenocephalides canis*	10 (50)	4/7
	Cat	8	27	21 *C. felis*, 6 *A. erinacei*†	8 (30)	3/8
Münster	Dog	3	9	6 *C. felis*,† 3 *A.erinacei*†	5 (56)	3/3
	Cat	1	3	*C. felis†*	3 (100)	1/1
Osnabrück	Dog	6	18	18 *C. felis*	0	0/6
	Cat	8	23	22 *C. felis*, 1 *Pulex irritans*	0	0/8
Rostock/Laage	Dog	5	15	12 *C. felis*, 3 *C. canis*	0	0/15
	Cat	1	3	*C. felis*	0	0/1
Schongau	Dog	5	15	12 *C. felis*,† 2 *Ceratophyllus gallinae*†	7 (47)	3/5
	Cat	5	15	13 *C. felis*,† 3 *C. gallinae*†	7 (47)	3/5

*gltA, citrate synthase. †Flea species positive for *gltA*.

Of 60 fleas positive for the *gltA* gene (for dogs and cats), only 2 were negative for the *ompA* and *ompB* genes. Sequencing analysis of the *gltA* gene for these 2 samples showed that 1 sequence (from *C*. *gallinae*) was 99% homologous with part of the *Rickettsia helvetica* *gltA* gene (AM418450.1) from an *Ixodes persulcatus* tick isolated in Russia; the other sequence (from *C*. *gallinae*) was 94% homologous with the *Rickettsia* sp. citrate synthase gene (U76908.1). Thus, we report *R*. *helvetica* in *C*. *gallinae* ticks.

Of the other 58 *gltA*-positive samples, 2 were positive for the *ompA* gene in the first round; 56 fleas were positive for the *ompB* gene. The 2 *ompA*-positive samples were sequenced, and sequences matched the *ompA* gene from *R*. *felis* (AJ563398.1; 99%–100% similarity). The 56 positive *ompB* samples were sequenced, and sequences matched with *ompB* gene from *R*. *felis* (CP000053.1; 98%–100% similarity). All hedgehog fleas (34 specimens) collected were infected with *R*. *felis*. Moreover, these 34 specimens were collected from 5 locations within a large area from Berlin (northeastern Germany) to Munich (southeastern Germany). Our findings indicate that *A*. *erinacei* may play a major role in the transmission of *R*. *felis* in Germany. Recent studies reported *R*. *felis* in 1 hedgehog flea in Portugal (*13*) and in 4 hedgehog fleas in Algeria (*8*).

## Conclusions

Our study confirms that *C*. *felis* remains the most common flea species infesting cats and dogs in Germany. Nevertheless, only 24 of 266 cat fleas collected were infected with *R*. *felis*. Infected cat fleas were found only in 4 of 11 studied sites, in contrast with a recent study in France, where *R*. *felis*–infected *C*. *felis* were present in all locations studied (*14*). In the 4 positive sites in Germany, 3 had positive *A*. *erinacei* specimens and 1 had positive *C*. *gallinae* (Table 2). In the other sites where no positive fleas where found, only *C*. *felis* was present either alone or in association with *P*. *irritans* and *C*. *canis* (Table 2).

Although *C*. *felis* seems to be the main vector of *R*. *felis*, our findings indicate that *A*. *erinacei* may be a vector for human flea-borne rickettsiosis in Germany. Because hedgehogs may act as a reservoir of pathogens (*15*), further studies will be conducted to investigate the role of hedgehogs and hedgehog fleas in maintenance and transmission of *R*. *felis* in Germany.
